# Injection drug induced septic embolism—A growing concern

**DOI:** 10.1016/j.radcr.2022.08.057

**Published:** 2022-09-15

**Authors:** Michael Kelson, Asaad Chaudhry, Andrew Nguyen, Sameh Girgis

**Affiliations:** a Department of Medical Sciences, Hackensack Meridian School of Medicine, 123 Metro Boulevard, Nutley, NJ 07110, USA; b Department of Internal Medicine, Jersey Shore University Medical Center, Neptune, NJ, USA

**Keywords:** Septic embolism, Pulmonary valve vegetation, Bacteremia, Opioids, Epidemic, Relapse

## Abstract

Septic pulmonary embolism is an obstruction of the pulmonary vasculature due to embolization of an infected thrombus. In many instances, the etiology is cardiac in origin, given the increased prevalence of intravenous drug users in the United States. This condition usually presents with fever, chest pain, dyspnea, and cough. In order to make the diagnosis, it is helpful to utilize tools like the modified Duke criteria when evaluating for infective endocarditis in the context of pulmonary emboli and septic shock. The gold standard method for establishing the diagnosis of this condition involves imaging modalities, including echocardiogram and computed tomography findings. This case report details a 36-year-old male with a history of drug abuse and hepatitis C, who was found to have an isolated vegetation on the pulmonic valve and septic pulmonary embolism. The patient experienced a rapidly deteriorating clinical course, however improved over the course of 2 weeks with supportive measures and appropriate antibiotic treatment. The purpose of this case report is to highlight the uncommon nature of pulmonary valve involvement in patients with infective endocarditis. Moreover, the goal of this report is to recognize the paralleled increase in septic pulmonary emboli with the rising incidence of patients using injectable opioids in the United States.

## Introduction

Septic pulmonary embolism (SPE) was previously diagnosed almost exclusively in patients with pelvic thrombophlebitis secondary to either postpartum uterine infection or septic abortion [1,2]. However, the risk factors for this condition have changed with time. In recent years, septic emboli have become increasingly prevalent in the United States, given the rise in incidence of people who use intravenous (IV) drugs [1], [2], [3]. Approximately 58% of SPE are attributed to IV drug use, followed by indwelling catheter (27%) and skin/soft tissue infections (13%) [4]. What was once thought to be a rare disease has now become a relatively common condition [1,4]. The most frequently cited clinical manifestations include fever (85.71%), chest pain (48.81%), dyspnea (48.21%), and cough (41.07%) [4]. As this condition is highly fatal, level of clinical suspicion for this diagnosis should remain high when individuals present with the abovementioned symptomatology [3], [4], [5]. Prior studies have shown that improper administration of antibiotics or delays in treatment could result in a 5-fold decrease in survival for people with SPE [6], [7], [8]. Therefore, prompt workup by appropriate diagnostic imaging and treatment with microbe-specific antibiotics can significantly reduce the risks of mortality as well as SPE-associated complications (eg, refractory shock, renal failure, life-threatening hemorrhage) [9,10].

## Case report

A 36-year-old man presented to the hospital in the summer of 2022 with several days of fever and dyspnea. The patient further reported fatigue, chills, arthralgia, myalgia, weakness, and a maculopapular rash on his anterior chest/back/abdomen. He was prompted to visit the emergency department (ED) due to persistence of his symptoms for several days with worsening right upper extremity pain and swelling. He denied any recent sick contacts, travel, hospitalizations, or surgeries. His past medical history was significant for anxiety, illicit drug use, and hepatitis C. He reported a significant amount of stress within the past week which caused him to relapse on heroine. His physical exam was significant for a 3 cm, erythematous, indurated region on the right forearm with serous drainage and noticeable track marks to the bilateral antecubital fossae. The patient was mildly hypoxic to 93% with tachycardia and dry mucous membranes. Lab workup was abnormal with the following results: leukocyte count 18.9 × 10^3^/μL, neutrophils 71%, bands 15%, d-dimer 20,647 ng/mL, erythrocyte sedimentation rate (ESR) 30 mm/h, C-reactive protein (CRP) 27.74 mg/dL, and blood cultures with growth of gram positive cocci in clusters, indicative of *Staphylococcus aureus* bacteremia.

**Fig. 1:**
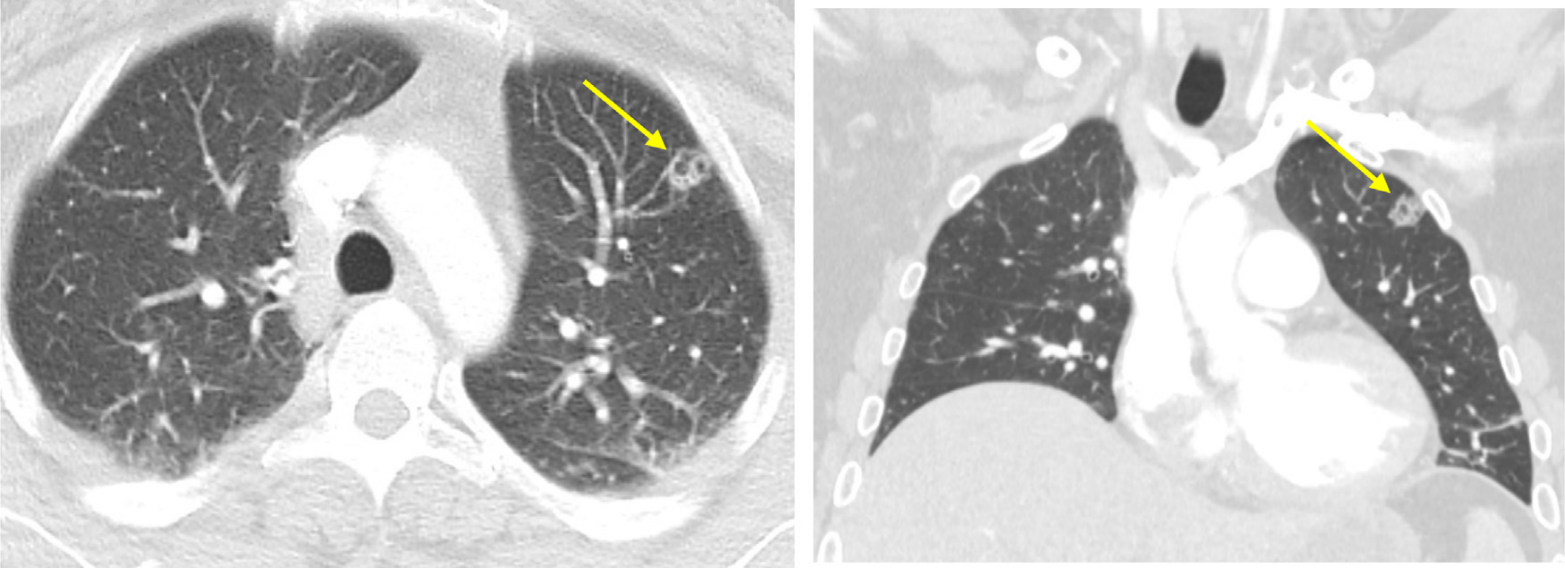
Axial (A) and coronal (B) images of CT chest show multiple bilateral pulmonary nodules, some of which demonstrate central cavitation (yellow arrow).

**Fig. 2:**
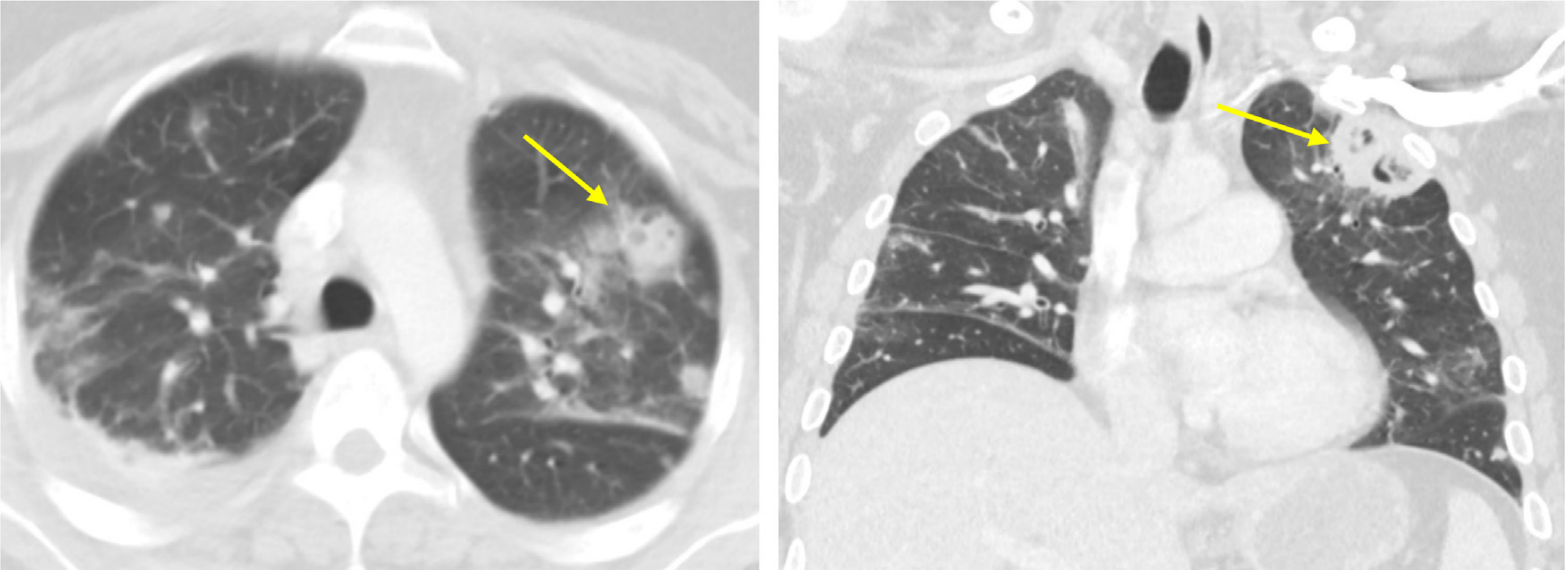
Axial (A) and coronal (B) images of CT scan demonstrate numerous areas of cavitation. Several of the nodules are new and some have demonstrated interval enlargement (yellow arrow) when compared to the prior study (Fig. 1). The large cavitary nodule in the left upper lobe measures 3.0 × 3.1 cm.

**Fig. 3:**
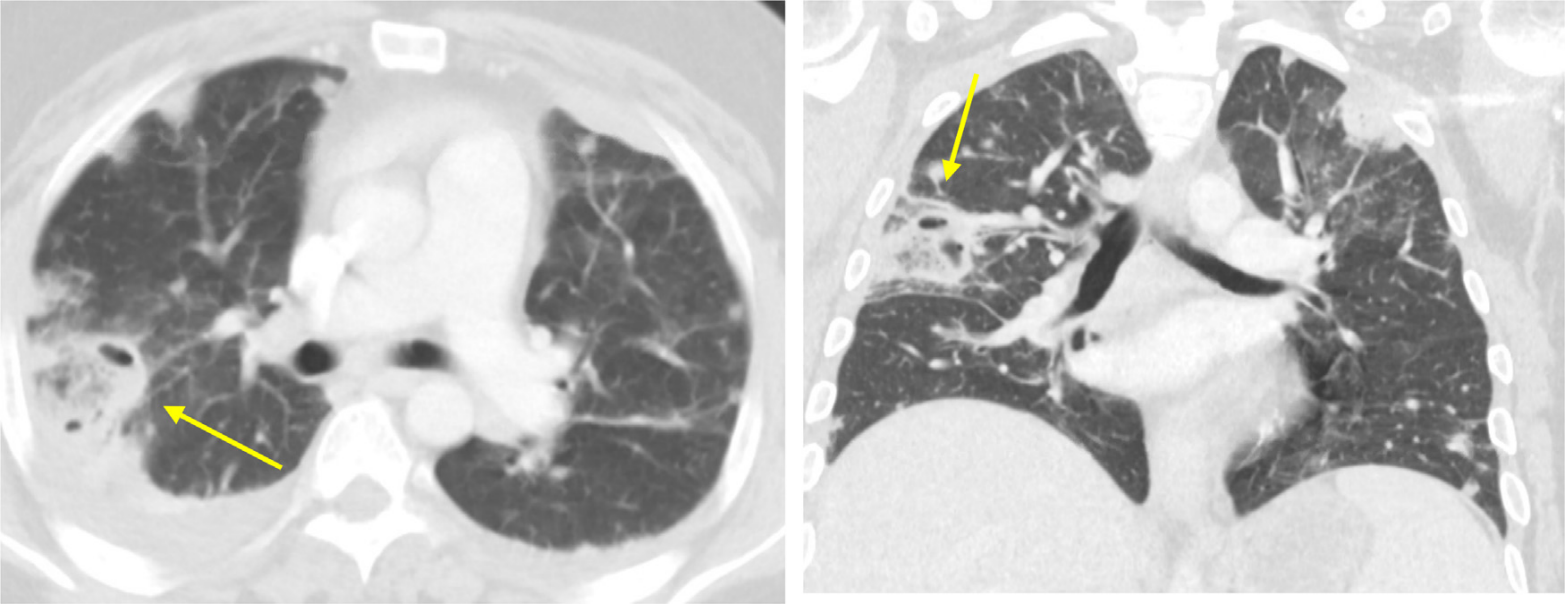
Axial (A) and coronal (B) images of CT scan show a large cavitary nodule in the right upper lobe, measuring 4.2 × 3.6 cm.

Electrocardiogram (ECG) portrayed normal sinus rhythm with occasional premature ventricular complexes (PVCs). Chest x-ray (CXR) showed no evidence of acute cardiopulmonary disease. Due to the patient's past medical history and presenting symptomatology, computed tomography (CT) of the chest with IV contrast was obtained. The findings demonstrated multiple bilateral pulmonary nodules, some of which exhibited central lucency, suggestive of septic pulmonary emboli (Figs. 1A and B). Filling defects were noticeable in the subsegmental branches of the right basal pulmonary artery. Also, small bilateral pleural effusions and underlying atelectasis can be visualized. Cardiology was consulted and transthoracic echocardiogram was performed. The study demonstrated right ventricular volume overload with an impaired relaxation filling pattern and a small echodensity on the pulmonary valve. In order to further assess for vegetations or abscesses, transesophageal echocardiogram (TEE) was conducted. The TEE findings were normal, with no evidence of masses or vegetations on the aortic, pulmonic, tricuspid, or mitral valves. Five days after the initial CT scan was performed, follow-up imaging studies were obtained (Figs. 2 and 3). As portrayed in Figs. 2 and 3, an increasing number of pulmonary nodules can be seen. Moreover, as depicted in Figs. 2A and B, we can see a large cavitary nodule in the left upper lobe of the lung, with interval enlargement evident when compared to the prior CT scan in Fig. 1. Thus, the patient's clinical condition was deteriorating rapidly over time.

The patient remained afebrile throughout most of his hospital duration. However, his white blood cell (WBC) count fluctuated significantly, with a peak level of 31.9 × 10^3^/μL. The patient was initially started on IV vancomycin 1.5 g QD (once-daily) and IV piperacillin-tazobactam (zosyn) 3.375 g Q8H (every 8 hours). With time, he developed hypotension and met diagnostic criteria for septic shock. The critical care team was consulted, and the patient received multiple fluid boluses to stabilize his vital signs. Zosyn was discontinued after blood cultures came back positive for *Methicillin-resistant Staphylococcus aureus* (MRSA). As per recommendations by the infectious disease team, vancomycin was discontinued and the patient was started on daptomycin (cubicin) 750 mg QD given the better outcomes associated with this medication for bloodstream infections due to MRSA. Unfortunately, the patient developed a new maculopapular rash on his bilateral lower extremities due to this antibiotic. The patient was switched to ceftaroline (teflaro) 600 mg Q8H with overall improvement of his clinical condition.

## Discussion

Septic pulmonary embolism is a life-threatening condition which can develop secondary to cardiac, exogenous, or peripheral endogenous sources [11,12]. In the context of IV drug users, a cardiac etiology is usually suspected due to bacterial inoculation of heart valves or pacemaker leads [3,13]. The epidemiologic data on right-sided endocarditis in adult patients references vegetations on the tricuspid valve as the underlying cause in the vast majority of cases while the pulmonic valve accounts for less than 2% of cases [14], [15], [16], [17]. As this is an uncommon clinical entity, isolated vegetations of the pulmonary valve require a high index of clinical suspicion with appropriate diagnostic testing in order to establish the diagnosis [11,18].

Laboratory workup for infective endocarditis (IE) may result in negative blood cultures up to 40% of the time [19], with some studies indicating false-negative results in over 70% of people [20,21]. Therefore, 2 or more blood cultures are usually obtained in order to increase the sensitivity of results (> 90%) when bacteremia is suspected [19,22]. Other non-specific indicators of IE include elevated inflammatory markers (ESR, CRP), normocytic anemia, and leukocytosis [23]. The modified Duke criterion, which is commonly used in everyday clinical practice, serves as a helpful diagnostic criterion for IE [24]. However, the gold standard method for establishing the diagnosis of this condition as well as related complications (eg, septic pulmonary embolism) involves imaging modalities, including echocardiogram and computed tomography findings [22], [23], [24], [25] (Table 1).

**Table 1:** Common CT scan findings in SPE.

	Frequency
1. “Feeding vessel” sign	90%
2. Peripheral nodules without cavitation	80%
3. Peripheral wedge-shaped opacities	75%
4. Peripheral nodules with cavitation	65%
5. Pleural effusion	65%
6. Lobar consolidation	40%
7. Lung abscess	30%
8. Ground-glass/hazy opacities	20%

*Source:* Chou et al. Septic Pulmonary Embolism Requiring Critical Care: Clinicoradiological Spectrum, Causative Pathogens and Outcomes. Clinics (Sao Paulo). 2016 Oct 1;71(10):562-569. doi: 10.6061/clinics/2016(10)02.

In the case of our patient, he developed infective endocarditis of the pulmonary valve, with subsequent embolization of the pulmonary vasculature and septic shock. The patient's clinical status deteriorated rapidly. However, with supportive measures and 1-2 weeks of appropriate antibiotic treatment, the medicine team was able to relieve this patient of his aberrant symptomatology. This case reminds us of the negative impact that the opioid epidemic is having on individuals in the United States. According to a study by Cicero et al., the use of heroin as an initiating opioid was 8.7% in 2005 [26]. In 2015, this number increased to 31.6% [26,27]. Moreover, just in the last 2 decades, the incidence of drug-overdose deaths has nearly quadrupled in this nation, with heroin-related mortality increasing by over 700% [27,28]. And as is the case with our patient, many individuals struggle with relapse after detoxification, with one study even reporting relapse rates up to 88% after 1-3 years of addiction medicine treatment [29,30]. Therefore, it is imperative that predictors for relapse in patients with substance use disorder are identified early so that appropriate, patient-tailored treatment strategies can be implemented to decrease hospital admission rates and subsequent complications of opioid use.

## Conclusion

Septic pulmonary embolism has increased in prevalence over the past several decades. The rise in incidence of SPE parallels the increased use of heroin across all demographics in the United States. As most studies corroborate, patients with a higher number of unsuccessful attempts to quit as well as longer durations of injecting are significant predictors of relapse. Thus, patients with these risk factors should receive comprehensive treatment strategies in order to alleviate their abuse potential.

## Patient consent

Written informed consent has been obtained from the patient to publish this paper.
